# 

**Published:** 1911-04-15

**Authors:** 


### BOOKS RECEIVED.

P. S. King and Son.
"Conference of Health Promoting Institutions."

The Universal Tutorial Press.

"Matriculation Directory."

W. B. Saunders and Co.
"Personal Hygiene and Physical Training." By Galbraith.

J. Nisbet and Co.
"Nisbet's Medical Directory, 1911."

### GIFTS AND BEQUESTS.

Miss Ellen Anne Cooke, of Cheltenham, has left £1,000 to the Cheltenham General Hospital.

Miss Elizabeth Cruse Cousins, of Clifton, has bequeathed £100 to the Bristol Royal Infirmary.

The Goldsmiths' Company has given £250 to the children's branch of the Metropolitan Convalescent Institution.

Mr. James Madder Tinline, of Teignmouth, Devon, who left estate of the gross value of £85,037, has bequeathed £100 to the Teignmouth Hospital.

A donkey, saddle, and harness have been presented to the Willesden District Council for the use of convalescent children in the grounds of the Isolation Hospital.

Miss Marianne Dudden, of St. Leonards-on-Sea, Sussex, has left the residue of her estate (apparently about £6,000) equally between the London Medical Mission and three other charities.

Mr. Edward Humphries Wiggett, of Bath, has left £200 to the Royal United Hospital, Bath, £200 to the Mineral Water Hospital, Bath, and £100 to the Eastern Dispensary, Bath.

Mr. Francis Barnitt, of Worcester, a member of the firm of Lea and Perrins, sauce manufacturers, who left estate valued at £118,401 gross, has bequeathed £250 to the Worcester Infirmary, unless he had given such a sum in his lifetime.

The London County Council will contribute £10,200 for the purchase of a site at Denmark Hill for a proposed hospital for the treatment of mental diseases. The site has been approved by the Commissioners in Lunacy, and Dr. H. Maudsley has promised to contribute £30,000 towards the cost of the hospital.

The proceeds of the gift of Messrs. Luce's Eau de Cologne, sold in the "Salon of Fragrance and Fair Women" at Harrods (Limited) last week, plus a special contribution and the sum received for admission, amounted to 1,000 guineas, which has been handed to the Middlesex Hospital in aid of the Prince Francis of Teck Memorial Fund.

A bequest of £4,000 enables the Council for the Promotion of the Higher Training of Midwives to contemplate the possibility of including in their building scheme at some future date a general hospital, which will materially assist in maintaining the high standard of midwifery training set up by the Woolwich Institution. The pioneer work carried on at Woolwich in this respect was commended by Sir Dyce Duckworth, M.D. (who lately inspected the Home on behalf of King Edward's Hospital Fund).

### JOTTINGS.

The After-Care Association has issued its report for 1910. Its most interesting section is the selected list of cases helped during the year, which illustrates practically the aims and successes of the society.

Princess Louise (Duchess of Argyll), the Duchess of Westminster, Lady Selborne, and Lady Ebury have issued an appeal on behalf of the Maternity Charity and District Nurses' Home, Plaistow.

The Birmingham and Midland Hospital for Women has agreed to become amalgamated with the Maternity Hospital and Lying-in Charity, the work of which it will take over at once. The Charity will not lose its identity.

Mr. H. F. Keep, chairman of the Committee of Management of the Queen's Hospital, Birmingham, reports that the experiment of employing the services of a lady almoner to check abuse in the out-patient department has been tried, and that he, personally, has little doubt as to the wisdom of extending it.

Lady Lewis, of 88 Portland Place, W., has lately presented the Royal Hospital for Incurables, Putney, with an Erard grand pianoforte for the new De Lancey Lowe room. The Hospital's appeal for a new organ for the assembly room is, we understand, likely to receive a favourable response at no distant date.

The Duke of Connaught, who is president of King's College Hospital, has given his patronage to the ladies' dinner in aid of the fund for the removal of the hospital to South London, and has forwarded a donation. The Earl of Selborne will preside at the dinner, which is to be held at the Connaught Rooms, Great Queen Street, W.C., on Thursday, May 4.

Princess Christian presided at the annual general meeting of the Ladies' Association of the Hospital for Women, Soho Square, last week. Earl Winterton (chairman of the committee) said that since its formation the Association had itself raised £3,600. They required £8,500 to pay off the mortgage debt and an additional annual income of £1,500 to meet the increased current expenses.

At the annual meeting of the Florence Nightingale Hospital for Gentlewomen, formerly the Hospital for Invalid Gentlewomen, Lisson Grove, N.W., Lord Waldegrave said that the name had been changed as a memorial to Miss Nightingale, who in 1853, fresh from Kaiserswerth, became its lady superintendent. The institution, not long started then, was languishing, and Miss Nightingale started the institution on a sound basis. It was from that hospital that in 1854, at only a week's notice, Miss Nightingale left with a band of nurses for Scutari.

### BUILDINGS CONTEMPLATED AND IN PROGRESS.

Bangor.—Mr. Bellis is architect for the workhouse infirmary building, estimated to cost £1,500.

Bradford.—Mr. F. Holland, architect to the Union Board, is engaged on extensions and additions to the Union Workhouse, Horton Lane, Bradford. Tenders are now being sent in.

Beaumaris.—The Port Sanitary Authority Joint Board invite tenders for the building of a floating hospital. Drawings, etc., may be inspected at the offices of the architect, Mr. Wm. Owen, M.I.Mun.E., at Menai Bridge. Tenders must be sent in before April 27.

Burford (Oxon.).—A new operating theatre is projected and plans may be seen at Fulbrook by builders desiring to tender. Mr. Wyndham Hughes is honorary secretary.

Chester.—Mr. Paul Waterhouse, F.R.I.B.A., has reported to the Board of the Infirmary on the suggested extension and improvement of the buildings. Mr. Waterhouse suggests a complete remodelling and the erection of an additional wing. Mr. Lockwood, architect to the Board, is preparing plans on the lines indicated.

Gateshead.—For the extension to the workhouse estimated to cost £26,000, Messrs. Newcombe and Newcombe, of Newcastle, are architects.

Kendal.—Tenders are now delivered for the alterations and additions to the hospital. Mr. J. F. Curwen, F.R.I.B.A., F.S.A., is architect.

London.—Mr. E. T. Hall, F.R.I.B.A., was employed in the Sir Harry Tyler wing just opened at the Homœopathic Hospital, Great Ormond Street. It contains three wards, a small operating room, *x*-ray rooms, etc.; the cost was about £30,000.

Old Windsor.—Plans of the alterations to the Union Workhouse may be seen at the offices of Messrs. Edgington and Spink, architects, Windsor.

Park Plewett (Hants).—Messrs Hine and Pegg are architects for the proposed well-water supply at the new asylum, Park Plewett, near Basingstoke. Tenders must be delivered not later than April 22.

### COMING EVENTS OF THE WEEK.

[Communications for this column must be sent to 29 Southampton Street, Strand, before noon on Wednesday.]

Tuesday, April 18.—London Throat Hospital, Gray's Inn Road, Lecture on "Method of Examination," by Mr. W. Stuart-Low, 3.45 p.m.

Friday, April 21.—A Sessional Meeting of the Royal Sanitary Institute will be opened by Mr. Walter F. Corfield, M.B., B.S., D.P.H., at the Town Hall, Colchester, at 7.30 p.m. Discussions will take place on "The Control of Pulmonary Tuberculosis."

### APPOINTMENTS VACANT.

Full particulars of these vacancies can be obtained on reference to our advertisement columns :

Charing Cross Hospital.—Resident Medical Officer.

Leicester Infirmary.—Assistant House Surgeon.

Liverpool Royal Southern Hospital.—Almoner and Out-Patients' Department Supervisor.

Prince of Wales' General Hospital, Tottenham.—Senior House Physician, Junior House Surgeon, Junior House Physician, Honorary Physician to *x*-Ray and Electrical Department.

Rugby Friendly Societies' Medical Institute.—Medical Officer.

Sheffield Royal Infirmary.—Junior Assistant House Surgeon.

West Bromwich District Hospital.—Assistant Resident House Surgeon.

"Encyclopédie Médicale" is the title of a new monthly medical bibliography on the plan of the *Annus medicus*, published at 32 rue Madame, Paris. The first number is a most excellent and exhaustive compilation, which will be found of much service to research workers and students. The subscription is 25 francs per year.

